# Microbiology of the Maxillary and Ethmoid Sinuses in Patients with Chronic Rhinosinusitis Submitted to Functional Endoscopic Sinus Surgery

**DOI:** 10.1016/S1808-8694(15)30058-6

**Published:** 2015-10-19

**Authors:** Josiane Faria de Aguiar Nigro, Carlos Eduardo Nazareth Nigro, Silvio Antonio Monteiro Marone, Richard Louis Voegels

**Affiliations:** a PhD in Otolaryngology, Assistant Professor of Otolaryngology at UNITAU.; b PhD in Otolaryngology, Assistant Professor of Otolaryngology at UNITAU.; c PhD and professor at FMUSP, Assistant Professor of Otolaryngology at HCFMUSP.; d PhD and Associate Professor of Otolaryngology at HCFMUSP.

**Keywords:** microbiology, chronic rhinosinusitis

## Abstract

Chronic rhinosinusitis microbiology studies show the presence of aerobe and anaerobe microorganisms, fungus and virus and their incidence vary according to each study. These studies guide us on choosing the most adequate antimicrobial agent to eliminate the infectious process, thus, helping in restoring rhinosinusal mucosa. **Study design:** Clinical prospective. **Aim:** This work aimed at studying the microbiology of the maxillary and/or ethmoid sinuses of patients with chronic rhinosinusitis and with indication of functional endoscopic sinus surgery. **Materials and methods:** During surgery, we collected secretion and/or fragments of maxillary and/or ethmoid sinus mucosa from 41 patients to perform Gram stain, fungus direct research, aerobe and anaerobe microorganism culture and fungus culture. **Results:** We identified the presence of aerobe microorganisms in 21 patients (51.2%), anaerobe microorganisms in 16 (39%) and fungus in 1 (2.4%). In the studied population, only 12 patients (29.2%) presented microorganisms considered pathogenic when analyzed together with the semi-quantitative leukocyte count. Staphylococcus coagulase-negative and Staphylococcus aureus were the most frequent microorganisms found, in 5 (12.18%) and in 4 (9.75%) patients respectively. **Conclusion:** This study reveals that Staphylococcus coagulase-negative and Staphylococcus aureus were the most frequent microorganisms isolated from patients with chronic rhinosinusitis.

## INTRODUCTION

Chronic rhinosinusitis (CRS) is defined as an inflammation of the nasal cavities mucosa and the paranasal sinuses for more than 12 weeks. The prevalence of chronic rhinosinusitis in the United States is estimated as present in 14% of the general population^1^. There is no epidemiological study in Brazil, but it is most likely similar to the American^2^ one. The sinus permeability might be involved due to anatomic alterations in the nasal cavity^3^, alterations in viscosity, volume and composition of nasal mucus and to alterations in the mucociliary transportation. These alterations might lead to mucus stasis, lower the local oxygen concentration with subsequent infection. The drainage ostium blockage is the main factor for the non-resolution of sinusitis in most patients^4^, ^5^.

The microbiological studies of CRS show the presence of aerobic and anaerobic microorganisms, fungi and viruses, varying the incidence according to each study. The microbiology studies of CRS guide towards choosing the most adequate antimicrobial to eliminate the infectious process, helping reestablish rhinosinusal mucosa homeostasis. The results of these studies vary according to the population studied, the mean of transportation used, time elapsed for processing the sample in the laboratory and the culture technique used^6^. We made this study in order to investigate the microbiology of the maxillary and/or ethmoid sinus of patients with CRS and with indication of functional endoscopic sinus surgery (FESS). During surgery we collected secretion and/or fragments of maxillary and/or ethmoid sinus mucosa.

## MATERIALS AND METHODS

This study was approved by the Ethics Committee of the University Hospital of the São Paulo University Medical School (HCFMUSP) and in all cases we obtained a signed consent either from the patient or the patient’s legal guardian.

We made a prospective study in 41 patients, 22 males and 19 females, with ages ranging from 13 to 75 years, diagnosed with CRS, seen at the otolaryngology outpatient ward at the HCFMUSP between June 2001 and December 2002. The sample calculated by the epidemiologist was of 41 patients with CRS after having gathered data on the incidence of microorganisms from the literature. All patients were above 13 years of age, with two major factors (nasal obstruction, nasal secretion, migraine, pain or sinus pressure and olfactory disturbance) or one major and two minor factors (fever, halitosis, cough and irritability) for longer than three months and with no improvement after clinical treatment with antibiotics (21 days), systemic steroids (10 days), nasal drops or decongesting agent when necessary, and nasal flush with 0.9% saline solution. Besides this, CT scan showed, in all patients, a blurring of one or more paranasal sinuses and of the meatal ostium complex. No patient had been using any antimicrobial agent 30 days prior to material collection. Patients with immunodeficiency, Killian polyp or malignant tumor of the nasal cavities were excluded. The same examiner selected the patients and collected the samples during surgery.

The 4 mm Karl Storz rigid endoscopes with 0° and 30° angle were immersed in glutaraldehyde for 30 min and then washed with sterile water, prior to surgery. Asepsis of the face and nasal vestibule were done with topical povidine. After placing surgical dressings, cotton patties embedded in xylocaine solution with adrenalin 1:2000 were placed for 10 min inside the nasal cavities for mucosa vasoconstriction. The endoscopic surgery was performed according to the Messerklinger technique. After opening the maxillary and/or ethmoid sinus, the cavity was seen and all secretion present was suctioned through a catheter connected to a syringe. The removal of a mucosal fragment was done through a Takahashi forceps, with care not to contaminate the sample with the nasal cavity mucosa.

The material collected was immediately prepared for proper transportation by the following: a sample of fragment was placed in thyoglicolate medium, to search for aerobic microorganisms; a sample of fragment was placed in Sabouraud medium to search for fungi; a sample of the fragment was placed in saline solution for direct study of fungi. A dish was prepared for bacterioscopy by the Gram method, and the secretion was placed in a sterile dry tube. The material was taken, in less than 30, to the microbiology laboratory for processing. Material was also sent for anatomopathological exam and was analyzed for the presence of fungi and Charcot-Leyden crystals.

## RESULTS

Among the 41 patients studied, 14 (34.1%) showed no growth of microorganisms, 12 (29.2%) showed the presence of only one microorganism, 11 (26.8%) showed the presence of two microorganisms and 4 (9.7%) showed three or more microorganisms. In 21 patients (51%) there was aerobic microorganisms growth, and in 16 (39%), anaerobic growth. In 1 patient (2.4%) there was fungus.

Coagulase-negative Staphylococcus was the most frequently found microorganism - in 5 patients (12.1%), whereas in 2 patients we found: Staphylococcus epidermidis. Staphylococcus aureus was found in four patients (9.75%). Enterobacter aerogenes, Haemophilus influenzae, Peptostreptococcus magnus, Peptostreptococcus sp. e Propionibacterium acnes were found in three patients (7.3%) each. In our case base only two patients had already undergone FESS, one of them showing growth of Staphylococcus aureus and coagulase-negative Staphylococcus; whereas the other showed Enterobacter aerogenes in the cultures. Table 1 depicts the microorganisms isolated and the total number of microorganisms, compared to the total number of positive cultures, the total number of cultures and the total number of patients.

**Table 1:** Microorganisms found in patients.

Microorganisms	From the total number of germs	Total number of Positive cultures	Total number of cultures	Total number of patients
	n = 52	n =29	n = 46	n = 41

**Aerobes**	28 (53,8%)	22 (75,8%)	22 (47,8%)	21 (51,2%)
Gram-positives	12 (23%)	11 (37,9%)	11 (23,9%)	11 (26,8%)
Corynebacterium sp	1 (1,92%)	1 (3,44%)	1 (2,17%)	1 (2,43%)
Staphylococcus aureus	4 (7,69%)	4(13,79%)	4 (8,69%)	4 (9,75%)
Staphylococcus coagulase-negativo	3 (5,76%)	3(10,34%)	3 (6,52%)	3(7,31%)
Staphylococcus epidermidis	2 (3,84%)	2 (6,89%)	2 (4,34%)	2 (4,87%)
Streptococcus viridans	2 (3,84%)	2 (6,89%)	2 (4,34%)	2 (4,87%)
Gram-negatives	16 (30,7%)	13 (44,8%)	13(28,2%)	12 (29,2%)
Enterobacter aerogenes	3 (5,76%)	3(10,34%)	3 (6,52%)	3(7,31%)
Enterobacter clocae	2 (3,84%)	2 (6,89%)	2 (4,34%)	2 (4,87%)
Escherichia coli	1 (1,92%)	1 (3,44%)	1 (2,17%)	1 (2,43%)
Haemophilus influenzae	3 (5,76%)	3(10,34%)	3 (6,52%)	3(7,31%)
Morganela morganii	1 (1,92%)	1 (3,44%)	1 (2,17%)	1 (2,43%)
Klebisiella pneumoniae	1 (1,92%)	1 (3,44%)	1 (2,17%)	1 (2,43%)
Proteus mirabilis	1 (1,92%)	1 (3,44%)	1 (2,17%)	1 (2,43%)
Pseudomonas aeruginosa	1 (1,92%)	1 (3,44%)	1 (2,17%)	1 (2,43%)
Serratia marcescens	3 (5,76%)	3(10,34%)	3 (6,52%)	2 (4,87%)
**Anaerobes**	23 (44,2%)	17 (58,6%)	17(36,9%)	16(39%)
Gram-positives	17 (32,6%)	15 (51,7%)	15(32,6%)	14(36,5%)
Clostridium hastiforme	2 (3,84%)	2 (6,89%)	2 (4,34%)	1 (2,43%)
Peptostreptococcus asaccharolyticus	1 (1,92%)	1 (3,44%)	1 (2,17%)	1 (2,43%)
Peptostreptococcus anaerobius	2 (3,84%)	2 (6,89%)	2 (4,34%)	2 (4,87%)
Peptostreptococcus indolicus	2 (3,84%)	2 (6,89%)	2 (4,34%)	2 (4,87%)
Peptostreptococcus magnus	4 (7,69%)	4(13,79%)	4 (8,69%)	3(7,31%)
Peptostreptococcus sp	3 (5,76%)	3(10,34%)	3 (6,52%)	3(7,31%)
Propionibacterium acnes	3 (5,76%)	3(10,34%)	3 (6,52%)	3(7,31%)
Gram-negatives	6(11,5%)	6 (20,6%)	6 (13%)	5(12,1%)
Bacterioides fragilis	1 (1,92%)	1 (3,44%)	1 (2,17%)	1 (2,43%)
Prevotella bivia	1 (1,92%)	1 (3,44%)	1 (2,17%)	1 (2,43%)
Prevotella denticola	2 (3,84%)	2 (6,89%)	2 (4,34%)	2 (4,87%)
Prevotella melanogenica	2 (3,84%)	2 (6,89%)	2 (4,34%)	1 (2,43%)
**Fungus**	1 (1,92%)	1 (3,44%)	1 (2,17%)	1 (2,43%)
Candida albicans	1 (1,92%)	1 (3,44%)	1 (2,17%)	1 (2,43%)

Of the 41 patients evaluated, from 5 we obtained secretion and a mucosal fragment; from 9 we obtained secretion aspirate and from 27 we obtained fragments of mucosa, adding up to a total of 46 samples for culture. There was a growth of microorganisms in 11 (58%) maxillary sinus samples and in 18 (66.6%) samples from the ethmoid sinus. From the secretion aspirate samples we obtained 8 (57%) positive cultures and from the fragment of mucosa we obtained 21 (65.6%) positive cultures. From the 14 secretion samples we observed growth of aerobe microorganisms in 8 (57%) and anaerobe in 4 (29%). Among the 32 mucosa fragment samples there was growth of aerobic microorganisms in 14 (43.7%), anaerobic in 13 (40.6%) and fungi in 1 (3.1%). Of the 19 maxillary sinus samples aerobic growth was observed in 9 (47%) and anaerobic growth was observed in 8 (42%). Of the 27 samples of the ethmoid sinus there was aerobic growth in 12 (44.4%), anaerobic in 9 (33.3%) and fungi in 1 (3.7%). There was no significant difference in relation to the type of microorganism between mucosal fragment and secretion aspirate or between maxillary or ethmoid sinuses.

The bacterioscopy was positive in 10 (21.7 %) of the 46 samples collected, showing the presence of cocci and/or bacilli, with the bacterial culture being positive in 9 samples. The direct fungi search was positive in 3 (6.5%) of the 46 samples collected, showing a presence of yeast, and, in one sample, the fungi culture was positive with the growth of Candida albicans. The anatomopathological exams of the mucosal fragments showed a chronic inflammatory process and the presence of fungus was not shown. No Charcot-Leyden crystals were found.

In the semi quantitative leukocyte count of the 27 patients with positive cultures, 15 (55.5%) showed rare and some leukocytes and 12 (44.4%) showed frequent and numerous leukocytes. Of the 41 patients with CRS, 12 (29.2%) showed possibly pathogenic microorganisms.

## DISCUSSION

The present study had as objective to verify the microorganisms present in cultures of secretion and/or mucosal fragments obtained from the maxillary and/or ethmoid sinus from patients with CRS, non-respondent to clinical treatment, with indications of FESS due to obstruction of the meatal ostium complex. The microbiological study is important to guide us towards choosing a specific antimicrobial, to augment the treatment of patients with CRS.

We observed a slight predominance of aerobic microorganisms in the aspirate, agreeing with previous studies^7^, ^8^. The microbiology floras of the maxillary and/or ethmoid sinus we observed were similar, in agreement with the studies by Van Cauwenberge e Ingels^9^. In mucosal fragments microbiology we found more frequently aerobic microorganisms agreeing with studies from various authors^10^, ^11^, ^12^, ^13^, ^14^.

In our study we used semi-quantitative leukocyte count to differentiate between the pathogenic germs and the contaminants. We admit that the presence of frequent (6 to 10 cells/field) and numerous leukocytes (more than 10 cells/field) in Gram stain indicate sinus infection; the presence of some rare leukocytes might suggest that the microorganism found is a contaminant^15^.

We haven’t seen growth of microorganisms in 14 patients (34%). In studies that use the same methods of sample collection the lack of bacterial growth varies between 17 and 60%^12^, ^13^, ^16^. In the present study, in 36.5% of the patients we saw polymicrobial growth agreeing with Ramadan^8^ who observes the same in 31%. We noticed a predominance of coagulase-negative Staphylococcus (12.1%) and Staphylococcus aureus (9.7%) agreeing with other authors^7^, ^10^, ^11^, ^12^, ^13^, ^14^, ^17^. The role of coagulase-negative Staphylococcus in the pathogenesis of CRS is still not clear^18^. It is present in the flora of the nasal and sinus mucosa of healthy individuals^14^, but it may play a role in the genesis of CRS because, in the antimicrobial sensitivity test, this germ showed resistance to various antibiotics^7^, and specific antibiotic therapy should be considered^19^. The pathogenic role of coagulase-negative Staphylococcus might be related to the presence of leukocytes in moderate or large quantities in the semi-quantitative count^20^. In this study, of the 5 patients with coagulase-negative Staphylococcus found, in 3 we found frequent and numerous leukocytes in the semi-quantitative count, but, in these patients, the culture was polymicrobial. Thus, in our study, it was not possible to attribute pathogenicity to coagulase-negative Staphylococcus.

We found enterobacteria in 26.8% of the patients, values close to the ones found by other authors^9^, ^10^. We know the importance of these germs in the GI tract, but more studies with the control group should be made to determine the role of these microorganisms in CRS.

In the microbiological studies of CRS the detection of anaerobes vary from 0% to 88%^11^, ^19^, ^21^. This variability might be explained by the difficulties found for its culture, despite following technical procedures so as to allow its growth. We found anaerobic agents in 39% of the patients, where Peptostreptococcus magnus (7.3%), Peptostreptococcus sp (7.3%) e Propionibacterium acnes (7.3%) were the most frequent. Kremer et al.^14^ haven’t found any strict anaerobes, and they believe the anaerobes present low importance in CRS. Our study detected, in a relevant way, anaerobic germs, probably due to the short time elapsed until de sample was dished.

In our study, fungi represented only 1.9% of the microorganisms, while in the study by Araújo they represent 14%^22^. Mitchell mentions that silicofluoride dye for direct fungi search and the PCR technique increase the sensitivity for fungi detection^23^. In our study we have not had access to these identification methods, which might explain why we found such a low positiveness for fungi.

The sample collection was done in an aseptic manner to avoid contamination, and the transportation of the material collected was done appropriately and rapidly sent to the laboratory for culture. We found 14 (34%) patients with negative cultures, but among the patients with positive cultures, 15 (55.5%) showed, by the semi-quantitative leukocyte analysis, that the microorganism found was possibly a contaminant. The quantitative analysis, in which we count the number of colonies present in the culture, is another way of evaluating the pathogenicity of the microorganism, thus the semi-quantitative analysis is more widely used. This way, out of the 41 patients with CRS, in 12 (29.2%) the microorganism isolated was possibly pathogenic.

All of our patients presented CRS non-respondent to the various clinical treatments with antimicrobial agents, CT scan showing obliteration of the meatal ostium complex, and for this reason were indicated FESS. Various authors^3^, ^5^, ^24^, ^25^ emphasize the relevant role of meatal ostium complex obstruction preventing the correct aeration and drainage of the sinus secretion in the origin of the inflammatory process of the paranasal sinus; other authors^4^, ^6^ point out the secondary role of pathogenic microorganisms in CRS. This being said, the finding of pathogenic microorganisms in only 29% of the patients was coherent, because, maybe, in this group of patients the cause for obstruction was the main reason for chronic rhinosinusitis. Thus, we believe that FESS is of high importance for these patients, because ventilation of the paranasal sinuses will allow for the reestablishment of the nasosinusal physiology. The knowledge of the CRS microbiology guides us towards choosing more adequate antibiotics to lower the inflammatory-infectious process of the nasosinusal mucosa.

## CONCLUSIONS

1. There was no growth of microorganisms in 14 patients (34%).
2. The most frequent microorganisms found in CRS patients were: coagulase-negative Staphylococcus in 5 patients (12.1%); Staphylococcus aureus, in 4 patients (9.7%); Haemophilus influenzae, Enterobacter aerogenes, Peptostreptococcus magnus, Peptostreptococcus sp. e Propionibacterium acnes, in 3 patients (7.3%) each.
